# Comparison of hybrid clones derived from human breast epithelial cells and three different cancer cell lines regarding in vitro cancer stem/ initiating cell properties

**DOI:** 10.1186/s12885-020-06952-9

**Published:** 2020-05-19

**Authors:** Sera Selina Fahlbusch, Silvia Keil, Jörg T. Epplen, Kurt S. Zänker, Thomas Dittmar

**Affiliations:** 1 Institute of Immunology, Center for Biomedical Education and Research (ZBAF), Witten/Herdecke University, Stockumer Str. 10, 58448 Witten, Germany; 2 Center for Biomedical Education and Research (ZBAF), Witten/Herdecke University, Stockumer Str. 10, 58448 Witten, Germany

**Keywords:** Cell fusion, Cancer stem cells, Breast cancer

## Abstract

**Background:**

Several physiological (fertilization, placentation, wound healing) and pathophysiological processes (infection with enveloped viruses, cancer) depend on cell fusion. In cancer it was postulated that the fusion of cancer cells with normal cells such as macrophages or stem cells may not only give rise to hybrid cells exhibiting novel properties, such as an increased metastatic capacity and drug resistance, but possibly also cancer stem/ initiating cell properties. Hence, hybrid clone cells (M13HS, M13MDA435 and M13MDA231) that were derived from spontaneous fusion events of human M13SV1-EGFP-Neo breast epithelial cells and HS578T-Hyg, MDA-MB-435-Hyg and MDA-MB-231-Hyg cancer cells were investigated regarding potential in vitro cancer stem/ initiating cell properties.

**Methods:**

CD44/CD24 expression pattern and ALDH1 activity of parental cells and hybrid clones was determined by flow cytometry. A colony formation and mammosphere formation assay was applied to determine the cells’ capability to form colonies and mammospheres. Sox9, Slug and Snail expression levels were determined by Western blot analysis.

**Results:**

Flow cytometry revealed that all hybrid clone cells were CD44^+^/CD24^−/low^, but differed markedly among each other regarding ALDH1 activity. Likewise, each hybrid clone possessed a unique colony formation and mammosphere capacity as well as unique Snail, Slug and Sox9 expression patterns. Nonetheless, comparison of hybrid clones revealed that M13HS hybrids exhibited more in vitro cancer stem/ initiating cell properties than M13MDA231 and M13MDA435 hybrids, such as more ALDH1 positive cells or an increased capacity to form colonies and mammospheres.

**Conclusion:**

The fate whether cancer stem/ initiating cells may originate from cell fusion events likely depends on the specific characteristics of the parental cells.

## Background

It is well-known that several physiological and pathophysiological processes depends on the biological phenomenon of cell fusion (for review see: [1, 2]). In cancer it was proposed that cell fusion might be associated with disease progression. Both in vitro and in vivo data revealed that tumor cells could fuse with normal cells, such as macrophages, fibroblasts and stem cells, thereby giving rise to hybrid cells that could exhibit novel properties, such as an enhanced metastatic capacity or an increased drug resistance [3–19]. Using a dual antibiotic selection strategy Lu and colleagues obtained hybrid cells that were derived from spontaneous fusion events of hygromycin-resistance and puromycin-resistant MDA-MB-231 breast cancer cells [18]. Gast and colleagues used differently labeled tumor cells (e.g., H2B-RFP) and macrophages (GFP) concomitant with time-lapse video microscopy to visualize the spontaneous fusion of the cells [4]. In addition to in vitro data various studies also showed that cell fusion events between tumor cells and normal cells do also occur in vivo. For instance, Jacobsen et al. showed that approximately 30% of the cells of a human breast adenocarcinoma xenograft-derived cell line had a mixed mouse and human karyotype including mouse/ human translocations [17]. Such cells, which most likely originated from spontaneous fusion events between the malignant human epithelium and normal host mouse stroma were tumorigenic with histopathologic features of malignancy [17]. Massive cell fusion events were observed in the tumorigenic intestine of an APC^Min−/−^/ROSA26 mouse that was surgically joined to a GFP mouse [15]. Transcriptome analysis showed that hybrids exhibited characteristics of both parental lineages, but also possessed a novel transcriptome profile that was different from either parental lineage [15]. Injection of ID8-RFP ovary carcinoma cells into GFP mice resulted in the origin of hybrid cells that were positive for both GFP and RFP [19], which further supports the hypothesis that tumor cells and normal cells could fuse in vivo. Similar data were reported by Gast et al. demonstrating that either injection of H2B-RFP B16F10 mouse melanoma cells into a GFP mouse or injection of H2B-RFP/Cre B16F10 cells into a R26R-stop-YFP transgenic mouse or injection of fl-dsRED-fl-GFP B16F10 cells into a Cre mouse resulted in the identification of tumor cell × normal cell hybrids [4]. Moreover, tumor cell × normal cell hybrids were not only found in the primary tumor, but also in the circulation of the mice [4] suggesting that hybrid cells might exhibit metastatic capabilities. In addition to animal studies tumor cell × normal cell hybrids were also identified in human cancers [4, 12, 19–22]. STR analysis of a primary tumor and a lymph node metastasis of a melanoma patient that received an allogenic bone marrow transplant revealed that cancer cells exhibited a mixed genome comprising of donor and recipient DNA [20]. Likewise, Gast et al. recently demonstrated that tumor cells obtained from female pancreatic ductal adenocarcinoma patients, which previously received a bone marrow transplant from a male donor were positive for the Y-chromosome [4] indicating that female cancer cells have fused with male bone marrow-derived cells. Moreover, Y-chromosome harboring pancreatic ductal adenocarcinoma hybrid cells were also found in the circulation of these patients, their presence furthermore associated with a poorer prognosis [4].

In addition to the hypothesis that cell fusion could give rise to hybrid cells exhibiting an increased metastatic capacity or an increased drug resistance Bjerkvig et al. postulated that also cancer stem/ initiating cells might originate from cellular hybridization events [23]. This could be either due to induction of genomic instability, which is a hallmark of cell fusion [16, 24–26] or due to the fusion of mutated stem cells and/or somatic cells [23]. Cell fusion-derived lung cancer cell × MSC hybrids acquired stem cell-like properties, exhibited increased expression levels of the stem cell transcription factors Oct4, Sox2, Nanog, Kif4 and Bmi1 and possessed an increased metastatic capacity [8]. Similar findings were reported for the fusion of hepatobiliary stem/ progenitor cells with hematopoietic precursor-derived myeloid intermediates [27], the fusion of umbilical cord MSCs with gastric cancer cells [28], and the fusion of MSCs with human breast cancer cell lines [14], which all gave rise to hybrids exhibiting cancer stem/ initiating cell-like properties.

In a previous study we have already demonstrated that M13HS hybrid clones, which originated from spontaneous fusion events between M13SV1-EGFP-Neo human breast epithelial cells and HS578T-Hyg human breast cancer cells, possessed cancer stem/ initiating cell-like characteristics such as an increased frequency of ALDH1 positive cells and an increased capability of forming colonies and mammospheres [29]. Here, additional human M13SV1 breast epithelial stem-like cell × human cancer cell hybrids were investigated regarding putative cancer stem/ initiating cell properties to prove whether this particular cell population could originate by cell fusion.

## Methods

### Cell culture

M13SV1-EGFP-Neo cells were generated and cultivated as described previously [29]. In brief, M13SV1-EGFP-Neo cells were derived from M13SV1 human breast epithelial cells (kind provided by James Trosko, Michigan State University, East Lansing, MI [30]) by stable transduction with the pEGFP-MCS vector, which contains a G418 resistance [31]. Cells were maintained in MSU-1 basal media (Biochrom GmbH, Berlin, Germany) that was supplemented with 10% FCS (Biochrom GmbH, Berlin, Germany), 1% penicillin/streptomycin (100 U/ml penicillin, 0.1 mg/ml streptomycin; Sigma-Aldrich, Taufkirchen, Germany), 5 μg/ml human recombinant insulin, 10 μg/ml human recombinant EGF, 10 nM β-estrogen, 0.5 μg/ml hydrocortisone, 4 μg/ml human transferrin (all chemicals were purchased from Sigma-Aldrich, Taufkirchen, Germany) and 400 μg/ml G418 (Biochrom GmbH, Berlin, Germany) [29]. Hygromycin resistant cancer cell lines (HS578T-Hyg, MDA-MB-435-Hyg, MDA-MB-231-Hyg) were derived from the appropriate parental cancer cell line (HS578T (HTB 126; LGC Standards GmbH, Wesel, Germany), MDA-MB-435 (HTB 129; LGC Standards GmbH, Wesel, Germany), and MDA-MB-231 (HTB 26; LGC Standards GmbH, Wesel, Germany) by stable transfection with the pKS-Hyg vector. All hygromycin-resistant cancer cell lines were maintained in the recommended media (HS578T: RPMI 1640 (Sigma Aldrich, Taufkirchen, Germany), MDA-MB-435 and MDA-MB-231: DMEM (Sigma Aldrich, Taufkirchen, Germany)) supplemented with 10% FCS (Biochrom GmbH, Berlin, Germany), 100 U/mL penicillin, 0.1 mg/mL streptomycin (Sigma-Aldrich, Taufkirchen, Germany) and 200 μg/mL hygromycin B (Pan Biotech, Aidenbach, Germany). A dual antibiotic selection procedure was used to isolate spontaneously formed M13SV1-EGFP-Neo × hygromycin resistant cancer cell hybrids [10, 11]. Parental cells (each 1 × 10^6^ cells) were co-cultured for up to 48 h in the appropriate cancer cell medium. Subsequently, media was replaced by cancer cell medium supplemented with 200 μg/ml hygromycin B (Pan Biotech, Aidenbach, Germany) and 400 μg/ml G418 (Biochrom GmbH, Berlin, Germany). Double resistant hybrid clones were individually picked and propagated for further characterization. All cells were cultured in a humidified atmosphere at 37 °C and 5% CO_2_. Parental cell lines and hybrid clones were assayed by short tandem repeat (STR) analysis [11, 32] and mycoplasma contamination was excluded by using the MycoAlert™ mycoplasma detection kit (Biozym; Hessisch Oldendorf, Germany).

### STR analysis

Genomic DNA was isolated using the Nucleo® Spin Tissue Kit (Macherey-Nagel, Düren, Germany) in accordance to the manufacturer’s instructions. The PowerPlex® 16 HS System was used for DNA typing [33]. In the system the following loci are co-amplified: D18S51, D21S11, TH01, D3S1358, Penta E, FGA, TPOX, D8S1179, vWA and Amelogenin, CSF1PO, D16S539, D7S820, D13S317, D5S818 and Penta D. In addition, a size standard was included as well as Taq DNA polymerase in the master mix. All 16 loci were amplified simultaneously from 100 pg of genomic DNA in a single tube and analyzed in a single injection. Automatic assignment of genotypes using the GeneMapper® ID and ID-X software were applied after separation of the PCR products on an Applied Biosystems 3500xL Genetic Analyzer (ThermoFisher Scientific, Bonn, Germany).

### Chromosome spreading

Chromosome spreading was performed as described previously [34]. In brief, parental cells and hybrids were maintained in the presence of 0.2 μg/ml colcemid (Sigma Aldrich, Taufkirchen, Germany) for four to six hours in a humidified atmosphere at 37 °C and 5% CO_2_. After harvesting the cells and one PBS washing step cells were carefully resuspended in 10 ml 75 mM KCl and incubated for 30 min. Cells were sedimented (160×g, 10 min) and the pellet was carefully resuspended in the remaining KCl solution. Subsequently, the cell pellet was fixated using methanol/ acetic acid solution (3:1; chemicals from Sigma Aldrich, Taufkirchen, Germany), whereby 10 ml of this fixation solution was added dropwise under continuous stirring to the cells. Then, the methanol/ acetic acid fixed cells were dropped onto a H_2_O wetted cover slip. Chromosomal DNA was visualized by Sytox Green staining (Invitrogen, Karlsruhe, Germany) as recommended by the manufacturer’s instructions and confocal laser scanning microscopy (Leica TCS SP5; Leica, Bensheim, Germany).

### Colony formation assay

The colony formation assay was performed as described previously [29]. In brief, cells (1 × 10^2^ per well of a 6-well plate) were cultured for up to 2 weeks in a humidified atmosphere at 37 °C and 5% CO_2_. Cells were washed with PBS (two-times) and fixed and stained with 6% glutaraldehyde and 0.5% crystal violet (reagents from Sigma-Aldrich, Taufkirchen, Germany) for 60 min at room temperature. Subsequently, 6-well plates were thoroughly washed with water and then air-dried. The relative colony formation capacity of the cells 6-well plates were determined by densitometric analysis of scanned 6-well plates using ImageJ (https://imagej.nih.gov/ij/download.html).

### Mammosphere formation assay

The mammosphere formation assay was performed as described previously [29]. In brief, individual wells of a 96-well plate were first coated with 50 μl poly-(2-hydroxyethyl-methacrylate) (poly-HEMA; 1.2% (w/v) in ethanol; Sigma-Aldrich, Taufkirchen, Germany) to avoid cell attachment to the well bottom. Before using, plates were kept for up to 7 days in the incubator at 37 °C to allow the ethanol to completely evaporate. Then, 5 × 10^2^ cells per well were seeded in mammosphere formation medium, which consists of 80% medium I and 20% medium II supplemented with 20 ng/mL EGF (human recombinant), 20 ng/mL FGF (human recombinant) and 0.39 μg/mL hydrocortisone (all supplements were from Sigma-Aldrich, Taufkirchen, Germany). Medium I: Methocult H4100 (40% (v/v); Stem Cells Technologies, Cologne, Germany) and DMEM (60% (v/v); Sigma-Aldrich, Taufkirchen, Germany). Medium II: MammoCult Human Medium (Stem Cells Technologies, Cologne, Germany) or DMEM/F12 (Sigma Aldrich, Taufkirchen, Germany). All mammospheres were grown for up to10 days. ImageJ (https://imagej.nih.gov/ij/download.html) was used for determining the diameter and the number of mammospheres, whereby only mammospheres with a diameter > 60 μm were considered for analysis.

### Flow cytometry

The relative CD24 and CD44 expression levels of the cells were determined by flow cytometry using a FACScalibur flow cytometer (Becton Dickenson, Heidelberg, Germany). Briefly, cells (1 × 10^5^) were washed once in PBS and resuspended in 100 μl PBS containing 1 μl CD24-PE (IgG2a; BD Biosciences, Heidelberg, Germany) and 1 μl CD44-APC (IgG2b, κ; BD Biosciences, Heidelberg, Germany). PE and APC conjugated isotype antibodies served as controls (Iso-IgG2a-PE; R&D Systems, Wiesbaden-Nordenstadt, Germany; Iso-IgG2b,κ-APC; BD Biosciences, Heidelberg; Germany). Samples were incubated for 15 min at 4 °C, washed again once in PBS and then analyzed by flow cytometry using the FL2-H and FL4-H detection channels.

The AldeRed™ assay (Merck Millipore, Darmstadt, Germany) was performed in accordance to the instruction manual and as described previously [29]. Briefly, PBS washed cells (2 × 10^5^) were resuspended in AldeRed 588-A substrate containing AldeRed assay buffer and then divided into two fractions. The specific ALDH1 inhibitor diethylamino benzaldehyde (DEAB) was added to one fraction, which served as a control. Both fractions were incubated for 30 min at 37 °C in the dark. Then, cells were sedimented (300×g, 5 min) and the pellet was resuspended in 500 μl of AldeRed™ assay buffer. Samples were stored on ice before FACS analysis. All FACS data were analyzed using WinMDI 2.9 (http://facs.scripps.edu).

### Western blot

PBS washed cells (2 × 10^5^ cells) were resuspended in 20 μl PBS. Subsequently, 10 μl 3× Laemmli sample buffer was added and cells and were lysed for 10 min at 95 °C. Samples were separated first by 10% SDS-PAGE and then transferred to an Immobilon PVDF nitrocellulose membrane (Merck Millipore, Darmstadt, Germany) under semi-dry blotting conditions. Depending on the used antibody membranes were either blocked with 10% (w/v) non-fat milk powder or 5% BSA in TBS-T (Tris-buffered saline with 1% (v/v) Tween 20). Sox9, Slug, Snail and β-actin were detected using the following antibodies: Sox9 (rabbit polyclonal; Santa Cruz Biotechnology, Heidelberg, Germany), Slug (rabbit monoclonal, clone C19G7; Cell Signaling Technology Europe B.V., Frankfurt am Main, Germany), Snail (rabbit monoclonal, clone C15D3; Cell Signaling Technology Europe B.V., Frankfurt am Main, Germany), β-actin (rabbit monoclonal, clone 13E5; Cell Signaling Technology Europe B.V., Frankfurt am Main, Germany), elf4E (rabbit polyclonal; Cell Signaling Technology Europe B.V., Frankfurt am Main, Germany), goat-anti-rabbit-IgG-HRP-linked (Cell Signaling Technology Europe B.V., Frankfurt am Main, Germany). Bands were visualized using the Aequoria Macroscopic Imaging System (Hamamatsu Photonics Germany, Herrsching am Ammersee, Germany) and the Pierce ECL Western blot substrate (ThermoFisher Scientific, Bonn, Germany) as described before [29, 35, 36].

### Statistical analysis

Statistical analysis was performed using an unpaired, two-tailed Student’s t-test.

## Results

### Generation of M13MDA231 hybrid clones

In accordance to M13HS hybrid clone cells [10, 11] and M13MDA435 hybrid clone cells [10, 11, 32] M13MDA231 hybrid clone cells were also derived from spontaneous cell fusion events. Human M13SV1-EGFP-Neo breast epithelial cells exhibiting stem cell properties and human MDA-MB-231-Hyg breast cancer cells were co-cultured and in total 14 hybrid clones were isolated using a dual antibiotic selection procedure. STR analysis revealed an overlap of parental alleles in all M13MDA231 hybrid clones (data not shown) indicating that hybrids truly originated from real cell fusion events, which is in accordance to M13HS and M13MDA435 hybrids [11, 32]. Table 1 summarizes the STR data of the parental cells and of those four hybrid clones, which have been randomly chosen for this study. In accordance to M13HS and M13MDA435 hybrid clones [10] a uniquely increased mean chromosomal count, which was nearly the sum of the mean chromosomal number of the parental cells, was also observed for M13MDA231 hybrid clones (Table 2).

**Table 1:** Genotypic analysis of M13SV1-EGFP-Neo × MDA-MB-231-Hyg hybrid clones

Locus	M13SV1- EGFP-Neo	MDA-MB- 231-Hyg	M13MDA 231–3	M13MDA 231–6	M13MDA 231–11	M13MDA 231–13
D3S138	120.00	128.16	120.00 128.24	119.87 128.17	120.00 128.16	119.87 128.16
TH01^a^	157.84	165.72 176.59	157.84 165.81 176.62	157.71 165.75 176.69	157.83 165.80 176.61	157.85 165.77 176.63
D21S11	215.20	223.25 237.22	215.12 223.26 237.28	215.19 223.25 237.28	215.25 223.26 237.22	215.18 223.27 237.21
D18S51	298.85 333.02	295.08 314.01	295.00 298.84 314.00 332.94	295.10 298.86 314.03 333.05	295.11 298.84 313.94 332.98	294.93 298.73 313.87 332.91
Penta_E	408.02 413.04	408.07	407.99 413.10	407.92	408.03	408.11
D5S818	130.50 134.53	134.53	130.44 134.57	130.38 134.53	130.37 134.53	130.37 134.65
D13S317	179.65	195.66	179.77 195.71	179.76 195.69	179.65 195.66	179.77 195.71
D7S820	220.22 224.18	220.22 224.28	220.12 224.19	220.21 224.07	220.13 224.07	220.26 224.19
D16S539	290.77 302.78	290.76	290.81	290.77	290.69	290.78
CSF1PO^b^	341.51 345.52	341.43 345.42	341.47 345.44	341.46 345.49	341.43 345.54	341.40 345.47
Penta_D	398.82 417.60	408.31 422.41	398.82 408.22 417.64 422.31	398.82 408.27 417.67 422.39	398.82 408.38 417.73 422.42	398.71 408.22 417.53 422.31
Amelo- genin	104.00	104.00	104.00	104.00	104.00	103.95
vWA^c^	138.43 150.23	142.21 158.19	138.31 142.20 150.23 158.20	138.43 142.20 150.10 158.07	138.30 142.22 150.17 158.19	138.42 142.31 150.30 158.21
D8S1179	222.20 234.16	226.17	222.21 226.16 234.15	222.20 226.17 234.13	222.22 226.05 234.08	222.23 234.10
TPOX^d^	280.87	268.86 272.91	268.98 272.91 280.81	268.89 272.81 280.84	268.98 273.03 280.81	268.98 272.92 280.88
FGA^e^	341.39	345.54 349.65	341.47 345.56 349.77	341.46 345.49 349.65	341.44 345.61 349.77	341.40 345.58 349.77

^a^ TH01: human tyrosine hydrolase; ^b^ CSF1PO: human c-fms proto-oncogene for CSF-1 receptor; ^c^ vWA: human von Willebrand factor; ^d^ TPOX: human thyroid peroxidase; ^e^ FGA: human alpha fibrinogen

**Table 2:** Mean chromosomal count of parental cells and M13MDA231 hybrid clone cells

Cell line	Mean chromosome number
M13SV1-EGFP-Neo	44 ± 14 ^a^
MDA-MB-231-Hyg	58 ± 18
M13MDA231–3	73 ± 21
M13MDA231–6	84 ± 17
M13MDA231–11	86 ± 17
M13MDA231–13	86 ± 20

^a^ Shown are the mean ± STD of at least three independent chromosome spreading experiments. Cells of different passages have been used

### Hybrid clone cells and parental cancer cell lines exhibit a similar CD44/CD24 expression pattern

**Fig. 1:**
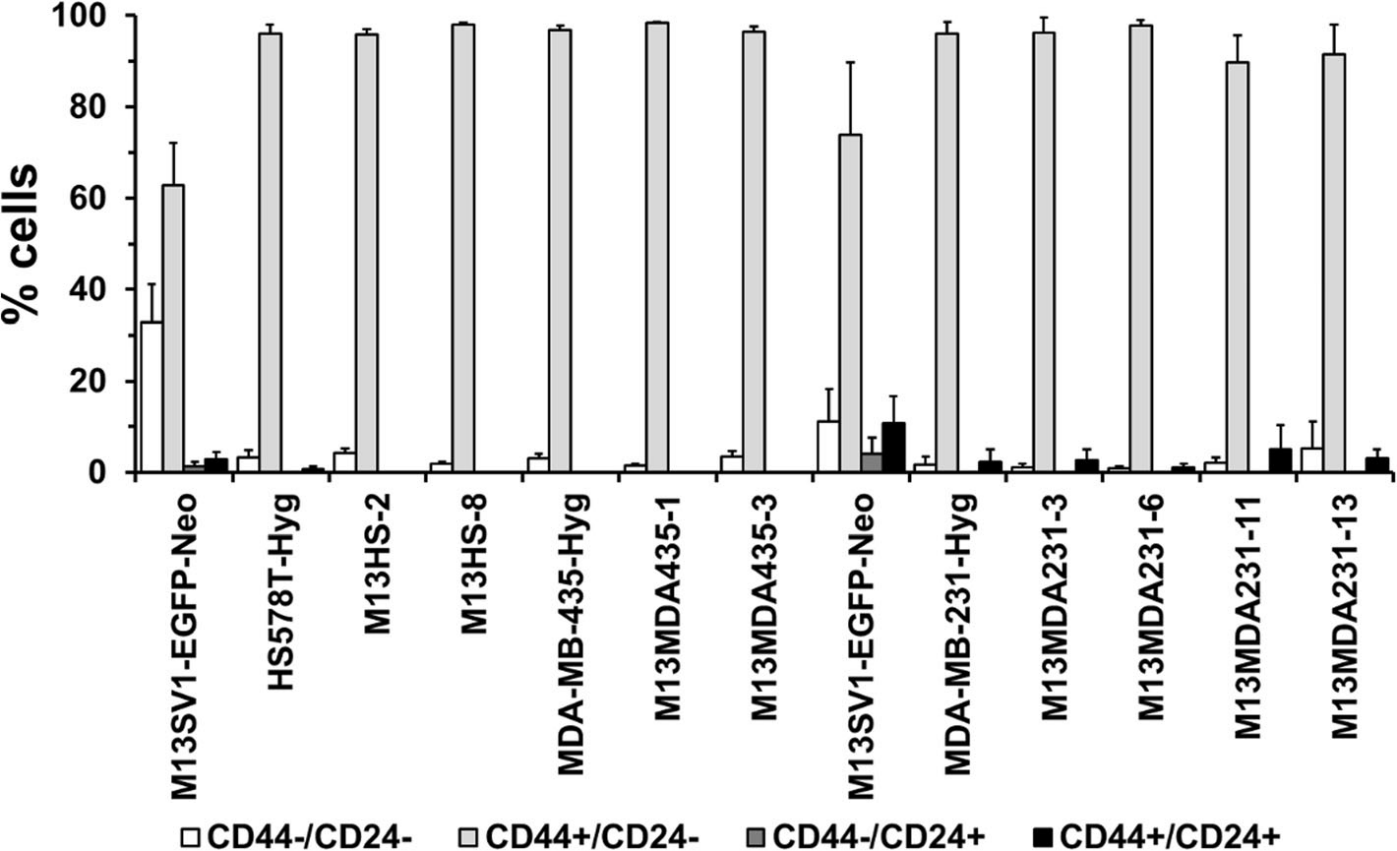
CD44/CD24 expression patterns. Cells were analyzed by flow cytometry using CD44-APC and CD24-PE antibodies in relation to isotype matched control antibodies. Two M13SV1-EGFP-Neo controls are shown here because M13HS and M13MDA435 hybrid clones and M13MDA231 hybrid clones were independently analyzed from each other. Shown are the means ± STD of at least three independent experiments

CD44 and CD24 have been suggested as suitable markers for the identification of breast cancer stem cells, whereby the phenotype of tumorigenic mammary tumor cells was determined as CD44^+^/CD24^−/low^ [37]. Hence, the CD44/CD24 expression pattern of parental cells and hybrid clones was analyzed by flow cytometry. In brief, flow cytometry data showed that parental cancer cell lines and all investigated hybrid clones exhibited a rather identical CD44/CD24 expression pattern with more than 95% of CD44^+^/CD24^−/low^ cells (Fig. 1).

### Hybrid clone cells possess a unique ALDH activity

**Fig. 2:**
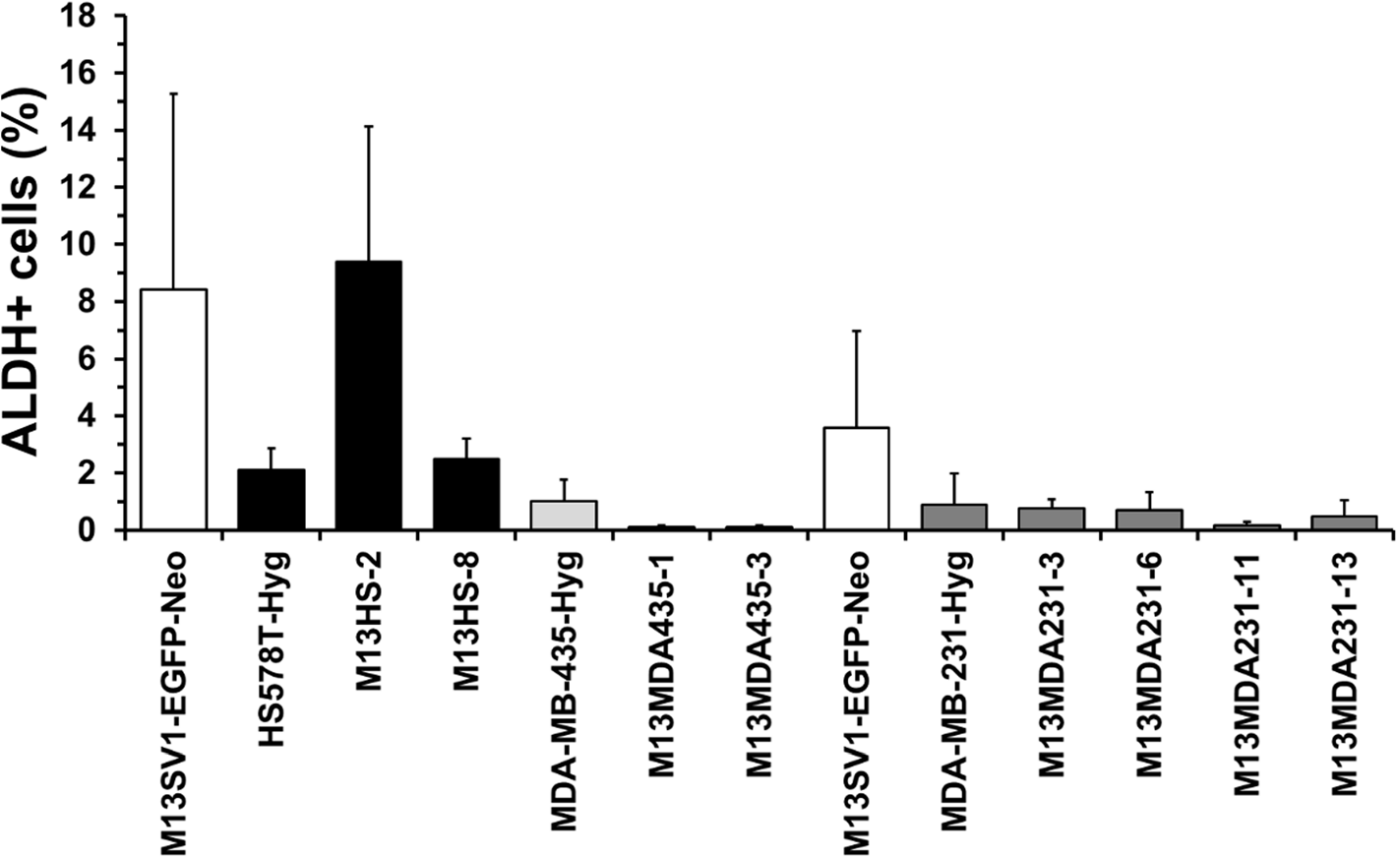
Parental cells and hybrid cells exhibit a unique population of ALDH positive cells. Two M13SV1-EGFP-Neo controls are shown here because M13HS and M13MDA435 hybrid clones and M13MDA231 hybrid clones were independently analyzed from each other. Shown are the means ± STD percentage of ALDH positive cells (ALDH data minus control data) of at least three independent experiments. Representative FACS data are presented in Supplementary Figure 1

An AldeRed™ assay was performed to determine the amount of ALDH positive cells within the population of parental cells and hybrid clones because ALDHs have been suggested as a suitable marker for both adult stem cells and cancer stem cells [38, 39]. Data for M13SV1-EGFP-Neo human breast epithelial cells, HS578T-Hyg human breast cancer cells and their hybrids M13HS-2 and − 8 were in accordance to previously published findings [29]. Both M13SV1-EGFP-Neo cells and M13HS-2 hybrids possessed an increased population of ALDH1 positive cells, whereas comparable levels of ALDH positive cells were observed for HS578T-Hyg cells and M13HS-8 hybrids (Fig. 2). However, in contrast to M13HS hybrids virtually no or even rather low levels of ALDH1 positive cells were found in M13MDA435 and M13MDA231 hybrids (Fig. 2).

### The colony formation capacity of M13SV1-EGFP-Neo × human cancer cell hybrids varies markedly

**Fig. 3:**
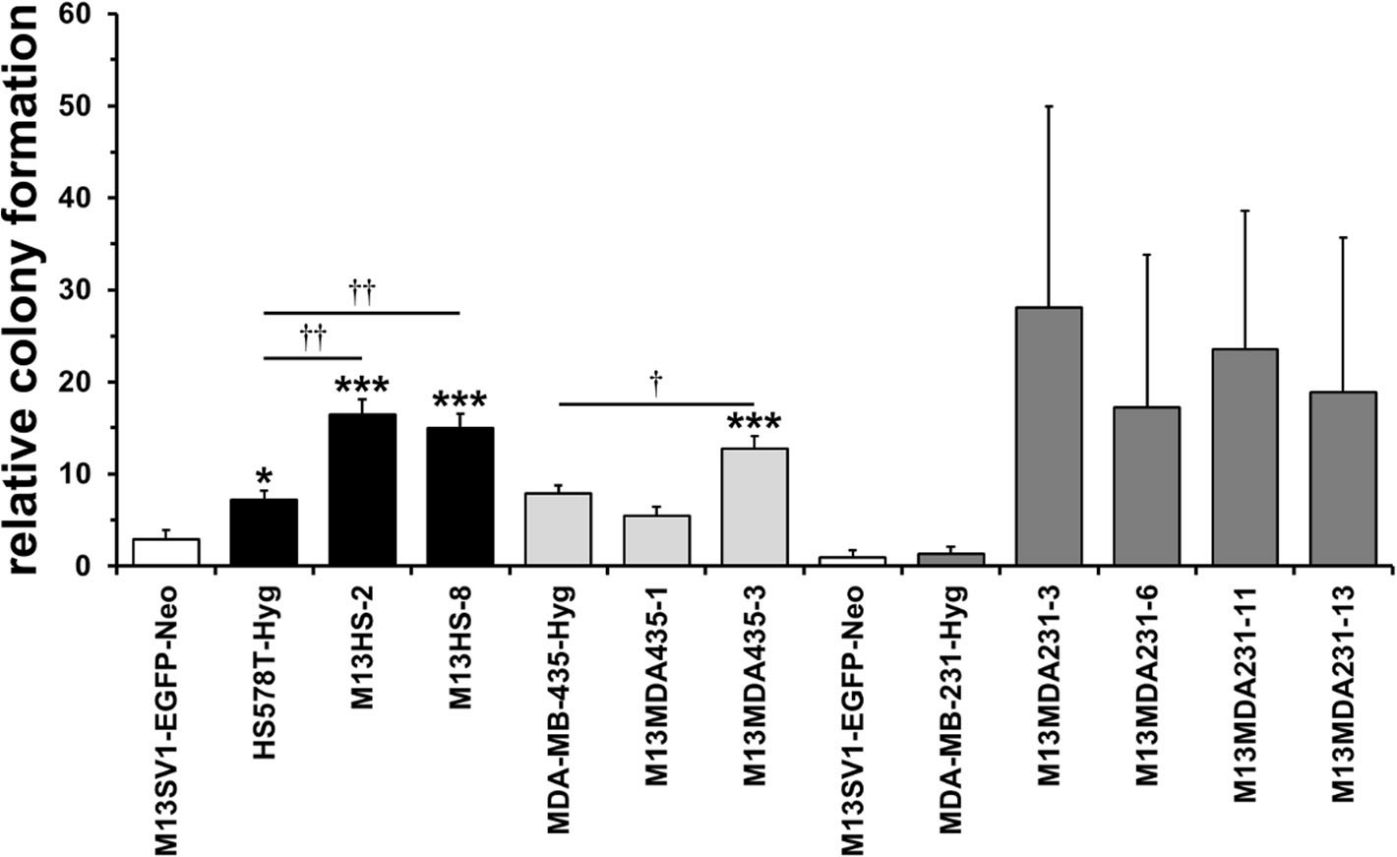
Hybrid clone cells possess an increased colony formation capacity. Two M13SV1-EGFP-Neo controls are shown here because M13HS and M13MDA435 hybrid clones and M13MDA231 hybrid clones were independently analyzed from each other. Shown are the means ± S.E.M. values of at least three independent experiments. * = *p* < 0.05, *** = *p* < 0.01 (in relation to M13SV1-EGFP-Neo human breast epithelial cells); † = p < 0.05, †† = p < 0.01

To investigate the parental cells and hybrid cells capacity to form colonies a colony formation assay was performed. M13HS-2 and − 8 hybrid clones possessed a similarly increased colony formation capacity as compared to their parental cell lines M13SV1-EGFP-Neo and HS578T-Hyg (Fig. 3), which is in agreement with previously published data [29]. In contrast, M13MDA435–1 and M13MDA435–3 hybrids exhibited a differential colony formation capacity. Significantly more colonies were formed from M13MDA435–3 hybrid clone cells, whereas the colony formation capacity of M13MDA435–1 hybrids was comparable to MDA-MB-435-Hyg cells (Fig. 3). A markedly, but not significantly increased colony formation capacity was observed for all investigated M13MDA231 hybrid clones (Fig. 3). Moreover, MDA-MB-231-Hyg human breast cancer cells exhibited a rather low colony formation capacity, which was comparable to that of M13SV1-EGFP-Neo human breast epithelial cells.

### Each M13SV1-EGFP-Neo × human cancer cell hybrid clone possesses a unique mammosphere formation capacity

**Fig. 4:**
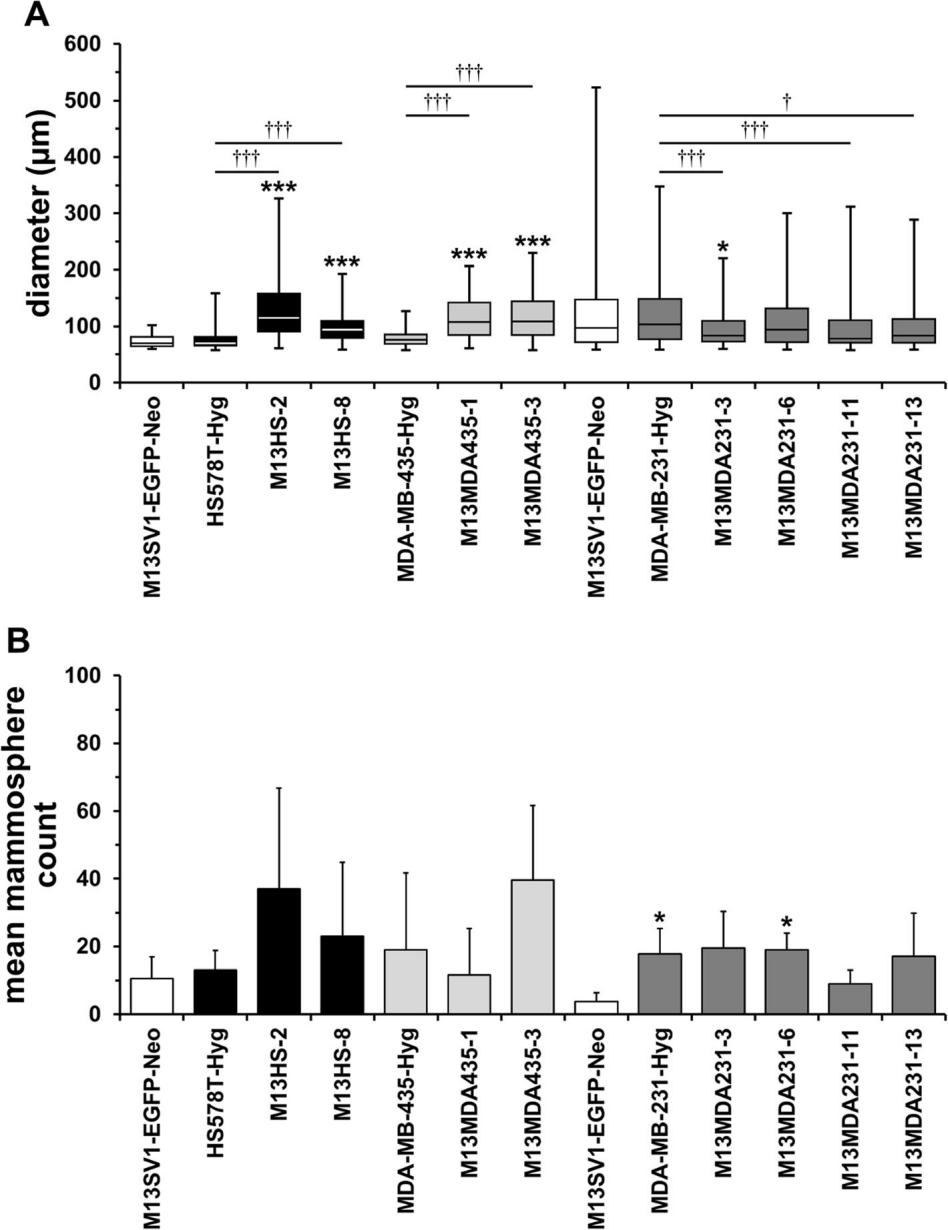
Hybrid clone cells possess a unique mammosphere formation capacity. **a**) BoxPlot diagram of the mammosphere size, whereby only mammospheres > 60 μm were included. **b**) Bar graph of the mean ± STD number of mammosphere (> 60 μm). Two M13SV1-EGFP-Neo controls are shown here because M13HS and M13MDA435 hybrid clones and M13MDA231 hybrid clones were independently analyzed from each other. Shown are the data of at least three independent experiments. * = p < 0.05, *** = p < 0.01 (in relation to M13SV1-EGFP-Neo human breast epithelial cells); † = p < 0.05, ††† = *p* < 0.001

Next, the hybrid and parental cells’ capacity to form mammospheres was investigated. In brief, each hybrid clone possessed a unique mammosphere formation capacity with regard to the size and the number of mammospheres formed. An increased number of mammospheres concomitant with a significantly larger diameter was observed for M13HS-2 and M13HS-8 (Fig. 4a,b). Significantly larger mammospheres were also derived from M13MDA435–1 and M13MDA453–3 hybrid clones, but only M13MDA453–3 hybrid cells showed an increased mammosphere formation capacity as compared to parental M13SV1-EGFP-Neo breast epithelial cells and MDA-MB-435-Hyg cancer cells (Fig. 4a,b). Interestingly, rather smaller mammospheres originated from M13MDA231–3, − 6, − 11 and − 13 hybrid clone cells as compared to M13SV1-EGFP-Neo breast epithelial cells and MDA-MB-231-Hyg breast cancer cells (Fig. 4a). Likewise, no increased mammosphere formation capacity was observed for these hybrids (Fig. 4b).

### Stemness factors are differentially expressed in M13SV1-EGFP-Neo × human cancer cell hybrids

**Fig. 5:**
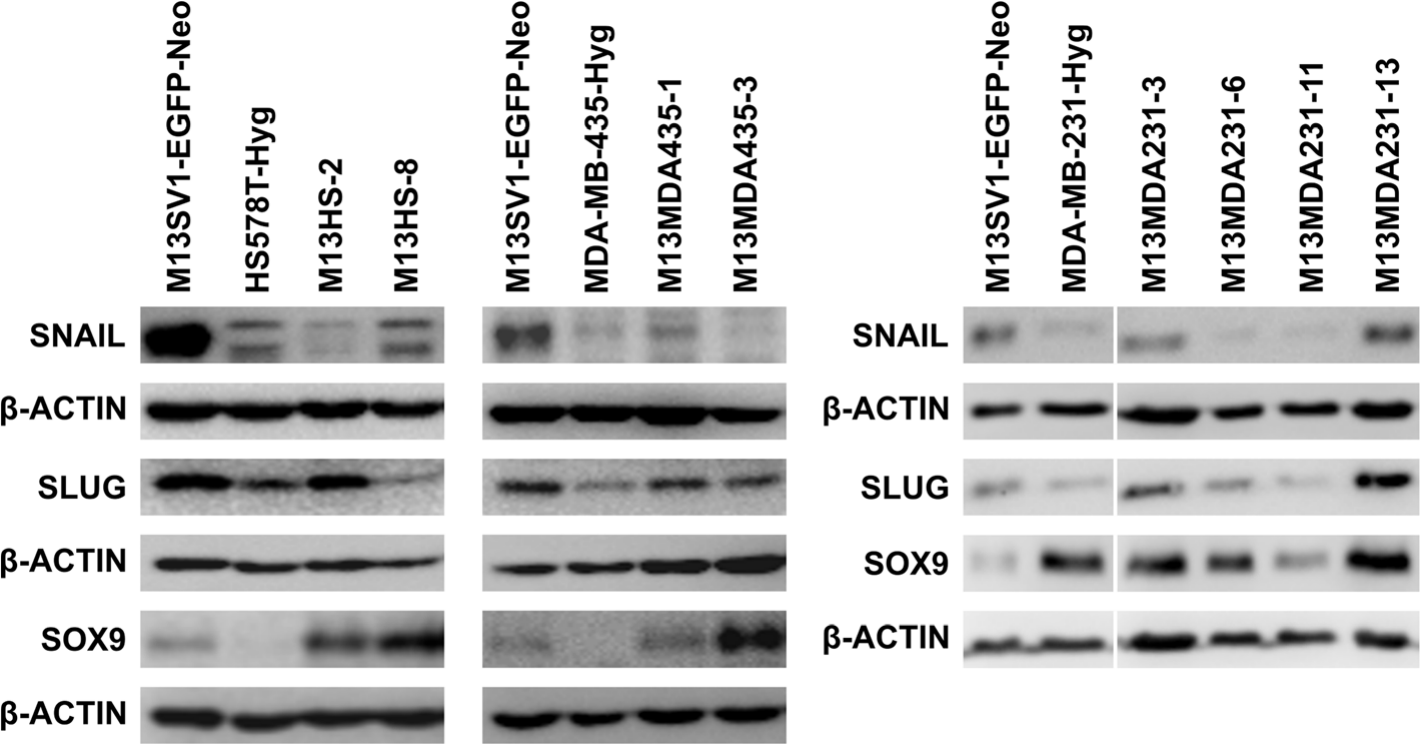
Hybrid clone and parental cells possess a different expression pattern of the stemness factors SNAIL, SLUG and SOX9. M13SV1-EGFP-Neo breast epithelial cells and MDA-MB-231-Hyg breast cancer cells express all three stemness factors, whereas both HS578T-Hyg and MDA-MB-435-Hyg cancer cells lack SOX9 expression. Hybrid clone cells are also positive for SNAIL. SLUG and SOX9, but each hybrid clone exhibits a distinct expression pattern. Shown are representative Western Blot data of at least three independent experiments. Full length blots are presented in Supplementary Figures 2, 3 and 4

Finally, Western blot studies were performed to estimate the relative expression levels of the transcription factors Slug, Sox9 and Snail in parental cells and hybrid clone cells, which have all been suggested as markers for mammary stem cells and tumor-initiating cells [40, 41]. M13SV1-EGFP-Neo human breast epithelial cells exhibiting stem cell properties were positive for all three transcription factors (Fig. 5), which is in accordance to previous findings [29]. Both HS578T-Hyg and MDA-MB-435-Hyg cancer cells co-expressed Snail and Slug, but lacked Sox9 expression (Fig. 5). In contrast, a markedly increased Sox9 expression, but rather low expression levels of Snail and Slug were observed in MDA-MB-231-Hyg breast cancer cells (Fig. 5).

In brief, co-expression of the three transcription factors was observed in all investigated hybrid clone cells (Fig. 5), whereby relative expression levels of single transcription factors varied among individual hybrid clones. For instance, high Slug, but low Snail expression levels were found in M13HS-2 hybrid clone cells, which was opposite to M13HS-8 hybrid cells (Fig. 5). Likewise, lower Sox9 levels, but higher Snail levels were found in M13MDA435–1 hybrid clone cells, which was opposite to M13MDA435–3 hybrids: Sox9 high, Snail low (Fig. 5). Comparable expression levels of all three transcription factors were observed in M13MDA231–3 and 13 hybrid clone cells, which was different to M13MDA231–6 and − 11 hybrids (Fig. 5). Marked Sox9, but rather moderate Snail and Slug expression levels were observed in M13MDA231–6 hybrid clone cells, whereas M13MDA231–11 hybrids exhibited the lowest Sox9, Slug and Snail expression levels of all analyzed hybrid clones (Fig. 5).

### Discussion

In the present study we investigated hybrid clones that were derived from spontaneous cell fusion events between M13SV1-EGFP-neo human breast epithelial cells exhibiting stem cell properties and three cancer cell lines regarding putative cancer stem/ initiating cell properties. The rationale of this work was attributed to the hypothesis that cell fusion may give rise to this particular cancer cell population [10, 23, 42]. A summary of the results is given in Table 3.

**Table 3:** Comparison of putative stemness characteristics of parental and hybrid clone cells

	CD44^**+**^/ CD24^**−/**^ low	ALDH^a^	CFA^b^	MA^c^ size	MA count	WB^d^ SNAIL	WB SLUG	WB SOX9
M13SV1-EGFP-Neo	++	+++	+/−	+/−	+/−	++	++	+
HS578T-Hyg	+++	+	+	+/−	+/−	+	+	–
M13HS-2	+++	+++	++	++	++	–	++	++
M13HS-8	+++	+	++	+	+	+	–	++
MDA-MB-435-Hyg	+++	–	+	+/−	+	–	–	–
M13MDA435–1	+++	–	+	++	+/−	+/−	+	+
M13MDA435–3	+++	–	++	++	++	–	+	++
M13SV1-EGFP-Neo	++	++	+/−	+	+/−	+	+	+/−
MDA-MB-231-Hyg	+++	–	+/−	+	+	–	–	++
M13MDA231–3	+++	–	+++	+/−	+	+	++	++
M13MDA231–6	+++	–	++	+/−	+	–	+/−	++
M13MDA231–11	+++	–	+++	+/−	–	–	–	+/−
M13MDA231–13	+++	–	++	+/−	+	++	++	++

^a^ ALDH: ALDH1 activity; ^b^ CFA: colony forming assay; ^c^ MA: mammosphere assay; ^d^ WB: Western blot

We are aware that the MDA-MB-435 cancer cell line is subject to controversy whether it is a melanoma or a breast cancer cell line. Gene expression concomitant with genomic hybridization and microsatellite polymorphism profile analyses revealed similarities between MDA-MB-435 cells and M14 melanoma cells [43, 44] suggesting that MDA-MB-435 cells were likely of melanoma origin. However, MDA-MB-435 and M14 cells revealed a differential DNA hyper-methylation profile [45]. MDA-MB-435 cells rather shared similarities with HS578T and BT-20 breast cancer cells, whereas the DNA hyper-methylation profile of M14 cells was identical to SK-MEL2 and SK-MEL28 melanoma cell lines [45]. Moreover, Sellapan and colleagues demonstrated that MDA-MB-435 cells express mammary specific proteins such as β-casein, α-lactalbumin, EMA, keratin-19 and even milk lipids upon β-Heregulin stimulation, which is indicative for breast cancer cells like MDA-MB-231, SUM1315 or HBL100 rather than for melanoma cells [46].

Flow cytometry data showed that all parental cancer cell lines and hybrid clone cells exhibited the putative breast cancer stem/ initiating cell phenotype CD44^+^/CD24^−/low^, which has been demonstrated to determine a highly tumorigenic breast cancer cell population [37]. About 1000 CD44^+^/CD24^−/low^ or 100 CD44^+^/CD24^−/low^/ ESA^+^, respectively, breast cancer cells were capable to induce tumor formation in NOD/SCID mice, whereas no tumor growth was initiated from even 500.000 CD44^+^/CD24^+^ breast cancer cells [37]. However, different studies raised concerns whether the CD44^+^/CD24^−/low^ phenotype would be a good marker profile for predicting breast cancer stem/ initiating cells. For instance, Wright and colleagues reported that two distinct CD44^+^/CD24^−^ and CD133^+^ cells with cancer stem cell characteristics were identified in Brca1 breast tumors [47]. Both populations were highly tumorigenic – as few as 50 to 100 CD44^+^/CD24^−^ and CD133^+^ breast cancer cells induced tumors in NOD/SCID mice – and expressed stem cell associated genes, including Oct4, Notch1, Aldh1, Fgfr1 and Sox1 [47]. Likewise, even though more than 90% of basal-like breast cancer cell lines were CD44^+^/CD24^−/low^ this phenotype was not correlated with tumorigenicity [48].

The enzyme ALDH1 has been suggested as a more reliable marker for identification and characterization of both adult stem cells and cancer stem/ initiating cells [49]. In contrast to the CD44^+^/CD24^−/low^ phenotype, which is rather associated with basal-like breast cancer, ALDH1 positive cells were found in basal-like, luminal and HER2 breast cancers [38, 39]. Likewise, expression of the ALDH1 isoform ALDH1A3 in patient breast tumor samples was significantly correlated with tumor grade, metastasis and cancer stage suggesting ALDH1A3 as a novel prognostic marker for breast cancer stem/ initiating cells [50]. ALDH1 positive cells encompassed only a minor (about 1.2%), but highly tumorigenic population of CD44^+^/CD24^−/low^ primary breast tumor cells [39]. As few as 20 ALDH1^+^/CD44^+^/CD24^−/low^ cells generated tumors, whereas even up to 50,000 ALDH1^+^/CD44^+^/CD24^−/low^ cells did not [39]. Likewise, ALDH1 positive primary breast cancer cells exhibited an increased colony and mammosphere formation capacity [39]. However, our data indicate no clear correlation between the hybrid cells ALDH1 expression levels and their capacity to form colonies and/ or mammospheres. M13HS-2 hybrids contained a rather high fraction of ALDH1 positive cells, which is well correlated to the cells’ capacity to form colonies and mammospheres. In contrast, an enhanced colony formation and mammosphere formation capacity was also observed for M13MDA435–3 hybrid clone cells albeit the cells were ALDH1 negative. However, breast cancer cells that were derived from human breast cancer xenografts were used in the work of Ginestier et al. [39], whereas in this study secondary cell lines and secondary cell line-derived hybrid cells were investigated, which might be a possible explanation for the observed differences regarding mammosphere formation capacity and ALDH1 activity. Interestingly, literature data are conflicting regarding ALDH1 expression and activity in MDA-MB-231 breast cancer cells. Both immunocytochemistry and ALDEFLOUR® assay data suggested that ALDH1 is expressed and active in human MDA-MB-231 breast cancer cells [51, 52]. In contrast, other studies and our work revealed that neither ALDH1 protein expression (Western Blot) nor ALDH1 activity (ALDEFLOUR®/ AldeRed™ assay) was detectable in MDA-MB-231 breast cancer cells [38, 53, 54]. Manuel Iglesias and colleagues reported that the capacity of breast cancer cell lines to form mammospheres depends on E-Cadherin expression [55]. Cell lines lacking E-Cadherin expression, such as SKBR3, MDA-MB-231 and MDA-MB-435 rather formed cell clumps than mammospheres [55]. However, mammosphere formation capacity was induced in SKBR3 cells transfected with an E-Cadherin expression vector, whereas MCF-7 breast cancer cells lost the capability to form mammospheres upon E-Cadherin knockdown [55]. The finding that neither MDA-MB-231 nor MDA-MB-435 cancer cells formed mammospheres [55] is in agreement with our findings. However, both M13HS-2 and M13HS-8 hybrids [29] and M13MDA435–1 and M13MDA435–3 hybrids lack E-Cadherin expression (unpublished data), but were able to form mammospheres suggesting that the capability of breast cancer cell lines to form mammospheres does not only depend on E-Cadherin expression.

Recently, it was proposed that the transcription factors Slug and Sox9 cooperatively determine the stem cell state of both normal and malignant mammary cells [40]. Ectopic Sox9 expression in CD49f^high^CD61^+^ basal cells, which already expressed significant levels of endogenous Slug, yielded in mammary stem cells exhibiting a markedly increased organoid formation capacity [40]. In contrast, knockdown of Slug in basal cells with constitutive Sox9 expression was associated with a loss organoid-forming capacity [40]. Likewise, knockdown of either Slug or Sox9 greatly inhibited the tumor-initiating ability of MDA-MB-231 breast cancer cells, whereas usually non-metastatic MCF7ras breast cancer cells became highly metastatic when Slug and Sox9 were co-expressed [40]. More recent data also suggested an important role for Snail in the biology of breast cancer stem/ initiating cells [41]. Knockdown of Snail but not Slug induced mesenchymal-to-epithelial transition, leading to loss of Zeb1 and reactivation of E-Cadherin in MDA-MB-231 breast cancer cells, which was further associated with an attenuated primary tumor growth and a strongly suppressed metastatic spreading [41]. Likewise, Snail but not Slug knockdown was also associated with a diminished tumorsphere formation capacity of most human breast cancer cell lines including HS578T [41]. Western Blot data revealed that parental cell lines and hybrid clone cells exhibited a unique expression pattern of three transcription factors. However, a clear correlation between Sox9, Slug and Snail expression levels and the cells mammosphere formation capacity was not observed. For instance, rather high Sox9 and Slug, but low to moderate Snail expression levels were observed in M13HS-2, M13MDA435–3, M13MDA231–1 and M13MDA231–3 hybrid clones, but only M13HS-2 and M13MDA435–3 hybrid clones exhibited an increased mammosphere formation capacity. Hence, we conclude that the ability of breast cancer cells and breast cancer hybrid cells does not only depend on the expression of transcription factors, but also on other properties, which have to be elucidated in ongoing studies.

The rationale of this study was to investigate whether cell fusion could give rise to hybrid cells possessing cancer stem/ initiating cells properties. Therefore, hybrid clones derived from human breast epithelial cells exhibiting stem cell properties and three different breast cancer cell lines were investigated. Both HS578T and MDA-MB-231 breast cancer cell lines have been classified as triple negative B, whereas MDA-MB-435 cells were grouped as triple negative A [56]. Of course, all three cell lines differ markedly regarding several characteristics, such as the mean chromosomal number, chromosomal aberrations, epigenetic profile, overall gene expression pattern, etc., which is the reason for the observed differences between M13HS, M13MDA435 and M13MDA231 hybrids (inter-hybrid clonal diversity). Additionally, cell fusion itself is a potent inducer of genomic instability due to HST, which is the merging the parental chromosomes and their random segregation during cell division [23–26]. Moreover, HST is commonly associated with further chromosomal aberrations including translocation, deletions, losses of whole chromosomes and putatively even chromothripsis [23–26]. Each evolving hybrid cell fusion of two cell types results always in individual hybrid clones (intra-hybrid clonal diversity) because of the unpredictable and unique process of HST [23–26].

## Conclusions

In conclusion, assuming that cancer stem/ initiating cells could originate by cell fusion, it would have been expected that more hybrid clones would have possessed cancer stem/ initiating cell properties. However, as shown here M13HS, M13MDA435 and M13MDA231 hybrid clones varied markedly among each other. For instance, only M13HS hybrid clones exhibited an increased colony formation and mammosphere formation capacity as well as contained more ALDH1 positive cells as compared to M13MDA231 hybrids, which only possessed an increased colony formation capacity. In conclusion, the fate whether cancer stem/ initiating cells may originate from cell fusion events likely depends on the specific characteristics of the parental cells.

## Supplementary information

**Additional file 1.**

**Additional file 2.**

**Additional file 3.**

**Additional file 4.**

## Data Availability

The datasets used and/or analyzed during the current study are available from the corresponding author on reasonable request.

## Abbreviations

ARP2: Actin-related protein 2
ARP3: Actin-related protein 3
ALDH1: Aldehyde dehydrogenase
BSA: Bovine serum albumin
CCL2: cc-chemokine ligand 2
DC-STAMP: Dendritic cell specific transmembrane protein
EMA: Epithelial membrane antigen
ESA: Epithelial specific antigen
FCS: Fetal calf serum
HST: Heterokaryon-to-synkaryon transition
IL-4: Interleukin-4
IL-6: Interleukin-6
IL-8: Interleukin-8
M-CSF: Macrophage-colony stimulating factor
MMP9: Matrix metallopeptidase 9
MSC: Mesenchymal stem cell
OC-STAMP: Osteoclast stimulatory transmembrane protein
PVDF: Polyvinyl difluoride
RANKL: Receptor activator of NF-κB ligand
STR: Short-tandem-repeat
SDS-PAGE: Sodium dodecyl sulfate-polyacrylamide gel electrophoresis
TBS-T: Tris-buffered saline with 1% (v/v) Tween 20
