# Supplementary figures and images for: Comparison of hybrid clones derived from human breast epithelial cells and three different cancer cell lines regarding in vitro cancer stem/ initiating cell properties
Source: BMC Cancer. 2020 May 19;20:446. doi: 10.1186/s12885-020-06952-9 (PMC7236176; doi:10.1186/s12885-020-06952-9)

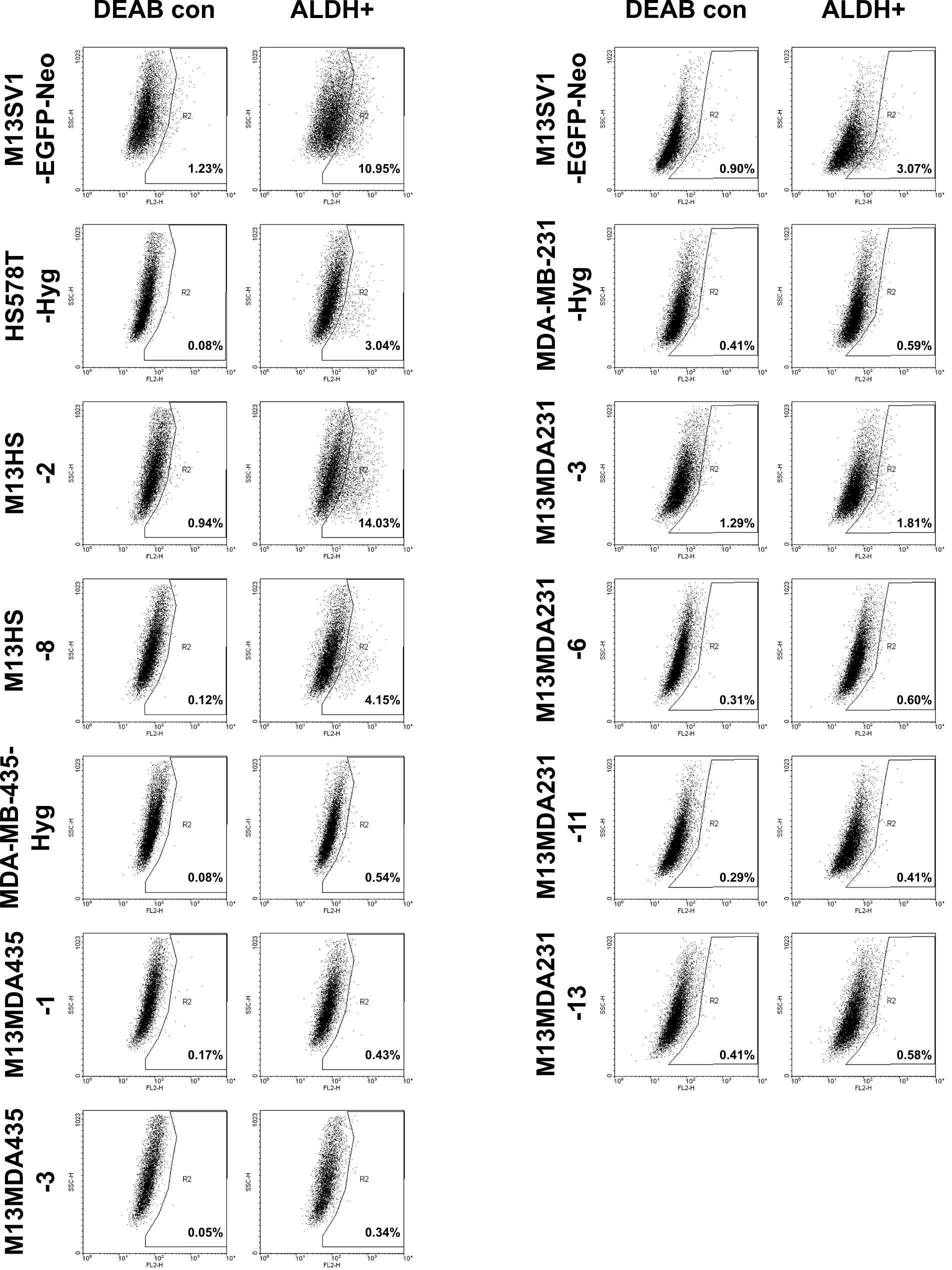

Supplement: Supplementary file 1 — Additional file 1. [file 12885_2020_6952_MOESM1_ESM.png]

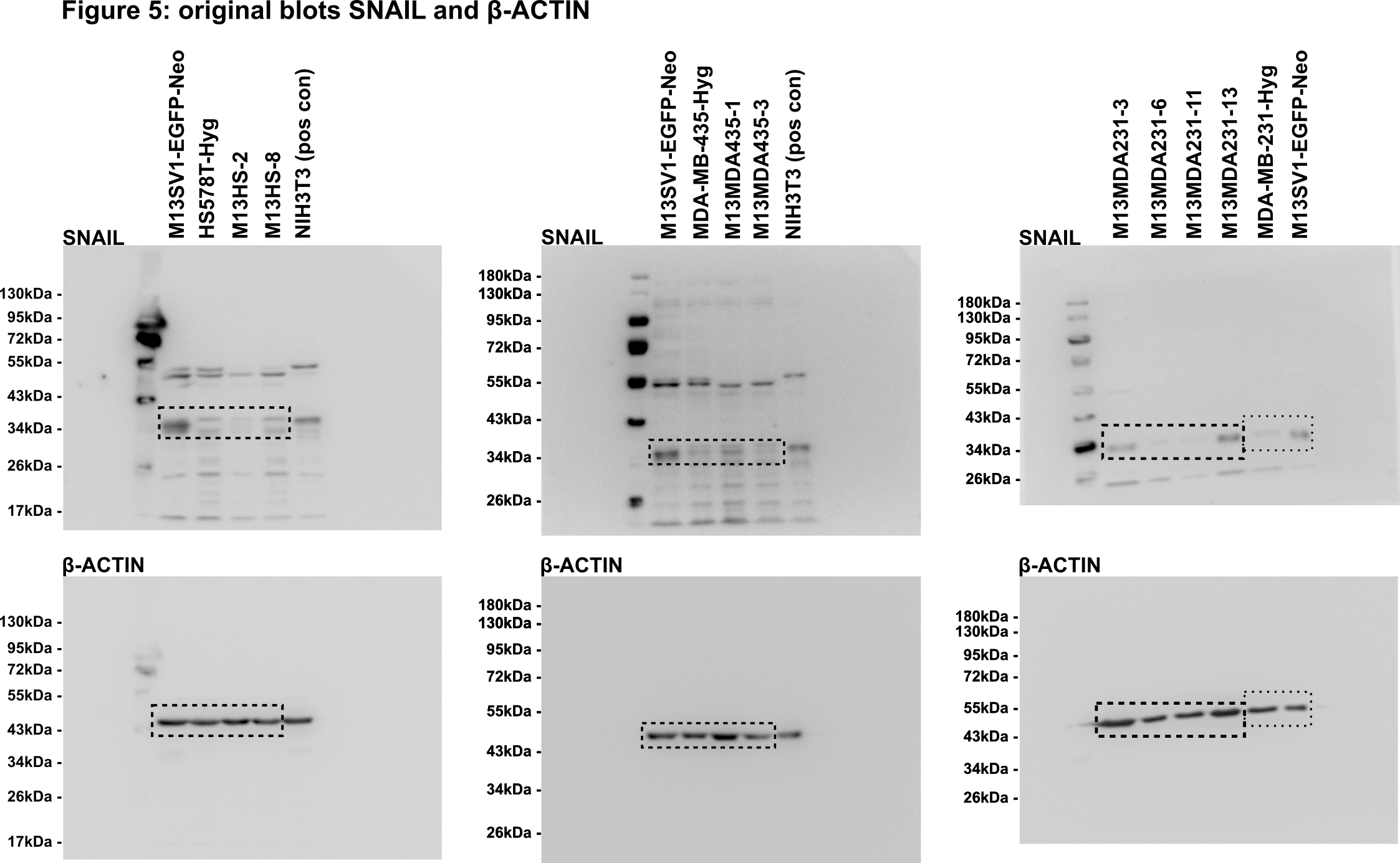

Supplement: Supplementary file 2 — Additional file 2. [file 12885_2020_6952_MOESM2_ESM.png]

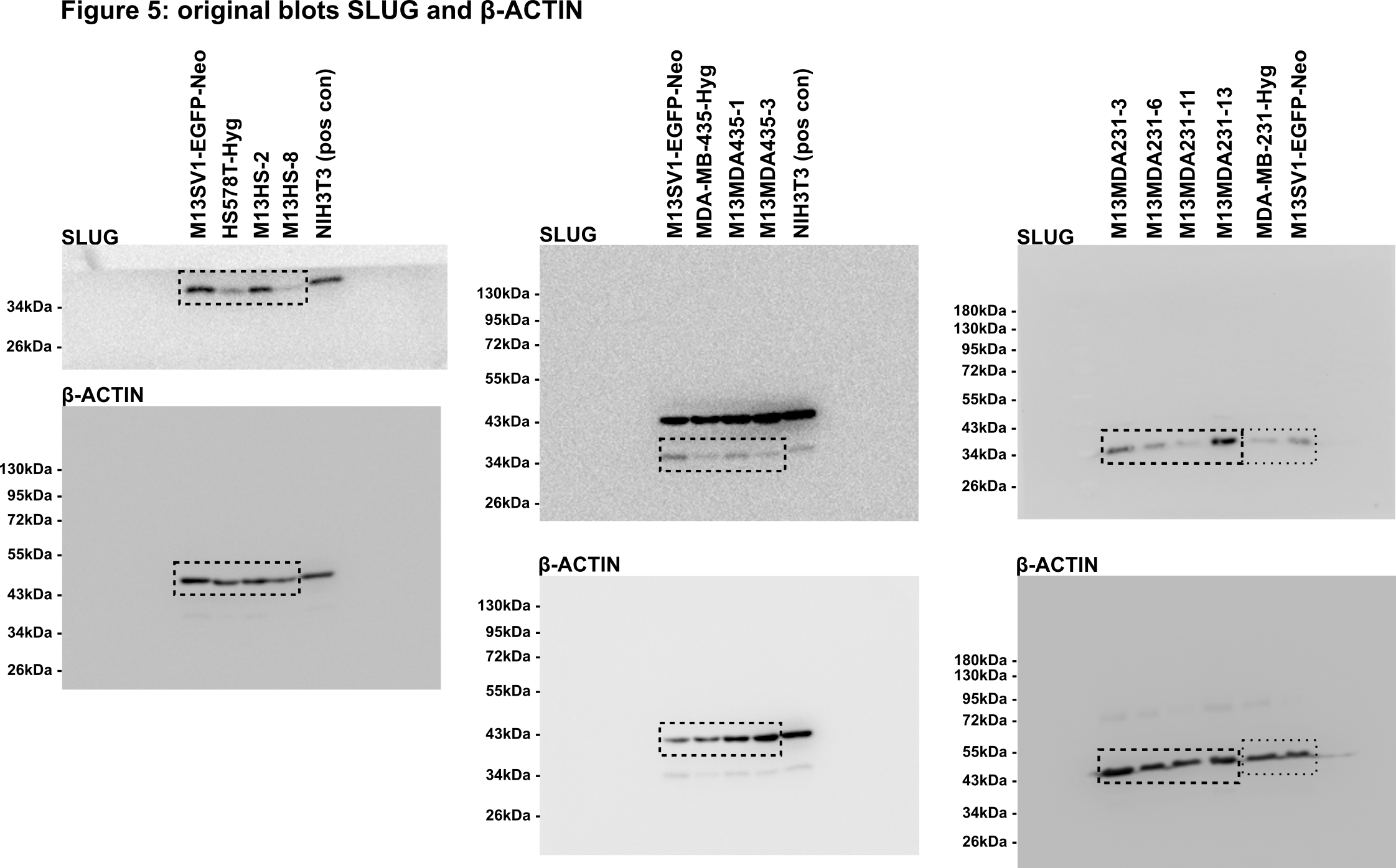

Supplement: Supplementary file 3 — Additional file 3. [file 12885_2020_6952_MOESM3_ESM.png]

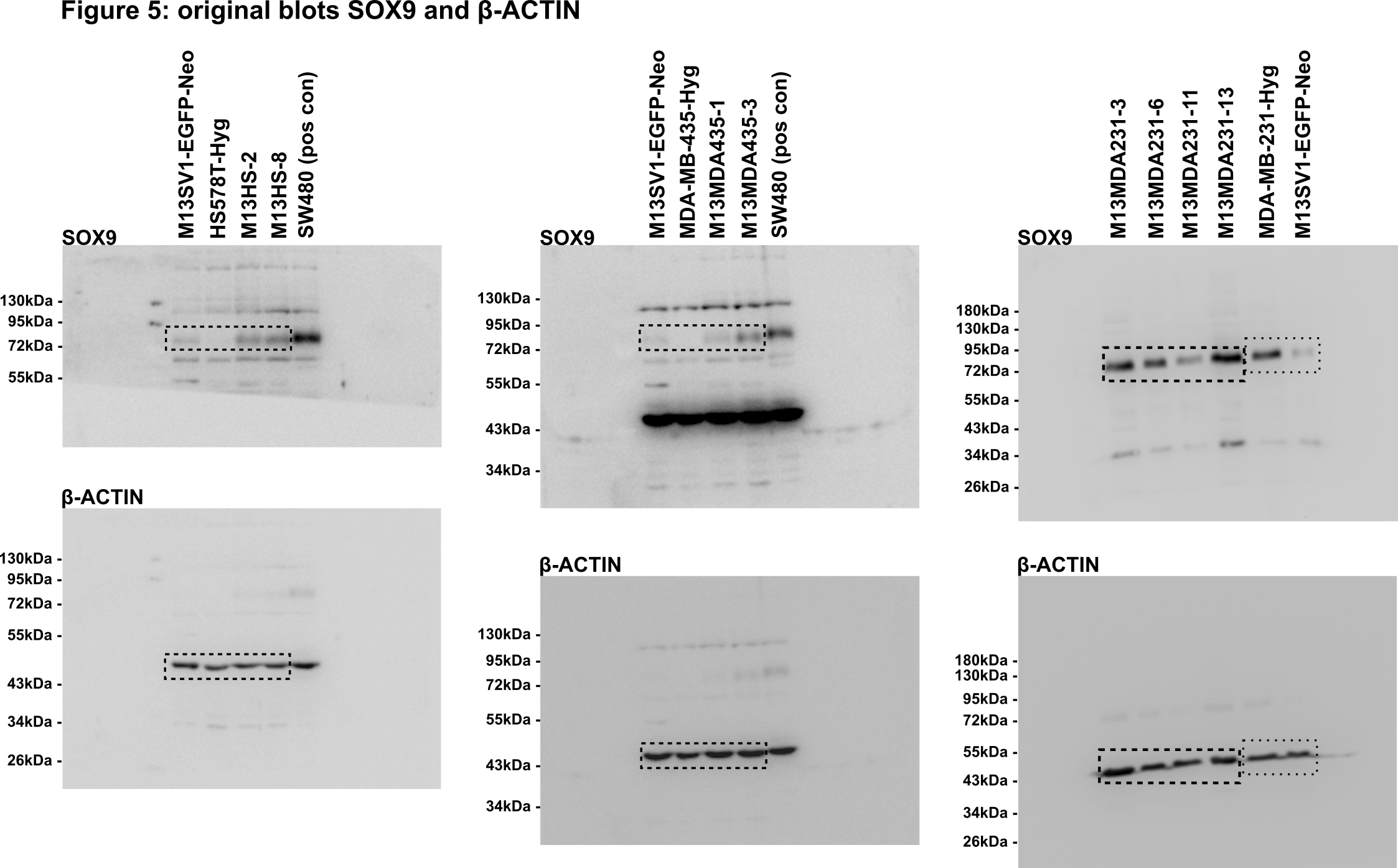

Supplement: Supplementary file 4 — Additional file 4. [file 12885_2020_6952_MOESM4_ESM.png]
